# Correlation between acetabular index at 3 and 12 months of age: a longitudinal radiographic study of 228 neonates treated for 6 or 12 weeks with the von Rosen splint for developmental dysplasia of the hip

**DOI:** 10.2340/17453674.2025.45043

**Published:** 2026-01-03

**Authors:** Adam SAND, Daniel WENGER, Henrik DÜPPE, Carl Johan TIDERIUS

**Affiliations:** Orthopaedics, Department of Clinical Sciences Lund, Lund University, Lund, and Department of Orthopaedics, Skåne University Hospital, Lund, Sweden

## Abstract

**Background and purpose:**

Developmental dysplasia of the hip (DDH) affects around 1.5% of newborns in Sweden with few late detected cases (0.12 per 10,000). The most common treatment for DDH in Sweden is with the von Rosen splint, with radiographs at 3 and 12 months of age. Little is known about the remodeling of acetabular dysplasia following treatment initiated in the neonatal period. We aimed to examine the correlation between the acetabular index (AI) at 3 and 12 months.

**Methods:**

We included 228 patients with early detected DDH with dislocatable hips (Barlow) and dislocated hips (Ortolani), treated with the von Rosen splint at Skåne University Hospital 2003–2019. The treatment length was 6 weeks for 96 children and 12 weeks for 132 children. We calculated the correlation between AI at 3 and 12 months using Pearson correlation (r) and the mean difference, both with 95% confidence intervals (CI).

**Results:**

The correlation between AI at 3 and 12 months was moderate, r = 0.43 (95% confidence interval [CI] 0.35–0.50), with changes in AI that differed widely. The mean AI was 23.9° (CI 23.5–24.3) at 3 months and 24.9° (CI 24.6–25.3) at 12 months with a difference of 1.0° (CI 0.6–1.3).

**Conclusion:**

The correlation between AI at 3 and 12 months was moderate, with non-clinical difference for both 6 and 12 weeks of treatment. The small increase in mean AI was most likely explained by a low AI at 3 months after 12 weeks of treatment.

Since the 1950s, practically every newborn child in Sweden is clinically examined by a pediatrician before leaving the maternity ward [1]. If hip dislocation or instability is detected, treatment is initiated immediately, most commonly using the von Rosen abduction splint within the first 2 weeks of life [2]. Since the inception of this nationwide clinical screening program, late detected cases of DDH have become rare; during 2000–2009, the incidence of hip dislocation diagnosed later than 2 weeks of age was 0.12 per 10,000 [2].

There are 2 principal methods to monitor DDH treatment in infants: radiography (AP pelvis) and static ultrasound, according to Graf [3]. Static ultrasound is used preferably in children below the age of 6 months while radiography can be used from approximately 3 months of age. The most common radiographic measurement is the acetabular index (AI),which has proven to be reliable and reproducible [4]. Although the term acetabular index is widely used, it is in fact an angle that is measured.

However, little is known about the remodeling of acetabular dysplasia following treatment initiated in the neonatal period. This is important when evaluating treatment effects and to determine the need for additional treatment, especially as we have shown that there is no difference in AI after 6 or 12 weeks of treatment at 12 months of age [5].

We aimed to examine the correlation between AI at 3 and 12 months. Our hypothesis was that AI will decrease between 3 and 12 months of age.

## Methods

### Study design

The study cohort has been described previously [5]. The cohort consists of newborns with a dislocated hip (Ortolani positive) or a hip that can be dislocated (Barlow positive) treated with the von Rosen splint and born at Skåne University Hospital, Sweden (Malmö and Lund). Patients were treated for 12 weeks between 2003 and 2010 in Malmö, and for 6 weeks between 2013 and 2019 in Lund.

### Diagnosis and treatment

**Figure 1:**
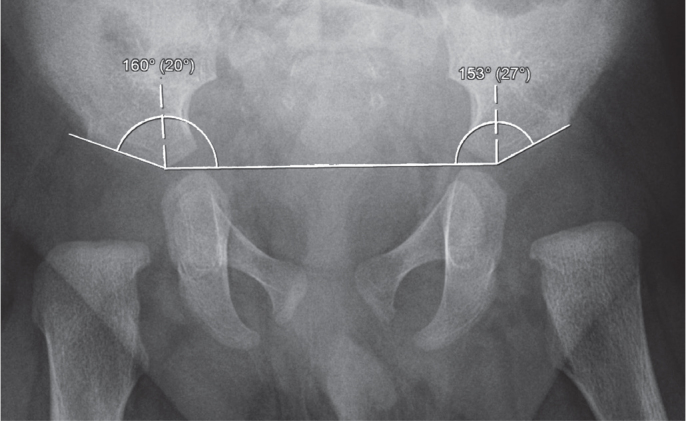
Acebatular index calculated at 3 months of age (20° right, 27° left). It is measured by drawing a horizontal line (Hilgenreiner) between the inferior portion of the iliac bones at the triradiate cartilages and measuring the angle formed between this line and a second line along the lateral acetabular roof.

At Skåne University Hospital, children with a dislocated hip or a hip that can be dislocated are referred to a pediatric orthopedic surgeon who initiates treatment [6,7]. Children with suspected hip instability are treated if > 25% of the femoral head can be subluxated, as assessed with dynamic ultrasound [8]. Treatment with the von Rosen splint was initiated within 2 weeks from birth in all children. The splint was worn day and night, and parents were informed not to remove it. All patients had weekly checkups and were bathed by nurses at our outpatient clinic. There was no difference in treatment between Barlow- or Ortolani-positive hips. We have shown that acetabular index (AI) at 12 months age does not differ between hips treated for 6 compared with 12 weeks [5]. AI at 3 and 12 months was measured on pelvic AP radiographs according to Tönnis (Figure 1) [9], and subgroup analysis of pathological and healthy hips was performed. The study is reported according to STROBE guidelines.

### Outcome measures

The primary outcome measure is the correlation between AI at 3 and 12 months. Secondary outcome measures are the difference in AI at 3 months of age between 6 and 12 weeks of treatment and the difference in AI between 3 and 12 months.

### Statistics

We used Pearson correlation (r) to calculate the correlation between AI at 3 and 12 months, expressed as r with 95% confidence intervals (CI). Interpretation of r values: < 0.10 was considered negligible, 0.10–0.39 was considered weak, 0.40–0.69 moderate, 0.70–0.89 strong, and ≥ 0.90 very strong [10]. Mean AI values and mean difference of AI values with 95% CIs are presented and significance tested by paired Student’s t-test. An intra-observer analysis was performed by calculating a 2-way random-effects model intraclass coefficient (ICC) with absolute agreement on 192 hips [11]. Data was handled according to the complete-case analysis method. Data analysis was performed using IBM SPSS Statistics version 29 (IBM Corp, Armonk, NY, USA).

### Ethics, data sharing, funding, use of artificial intelligence tools, and disclosures

The Swedish ethical review authority approved the present study (2020-04747), and parents gave verbal informed consent upon entry in the registry. The study was supported by grants from the Kockska Foundation, Swedish governmental funding of clinical research (ALF), and Stiftelsen för medicinsk vetenskaplig forskning inom ortopedi vid Malmö Allmänna Sjukhus. Pseudonymized study data can be obtained upon request, including ethical board approval. AI tools were not used. All authors declare that they have no conflicts of interest. Complete disclosure of interest forms according to ICMJE are available on the article page, doi: 10.2340/17453674.2025.45043

## Results

**Figure 2:**
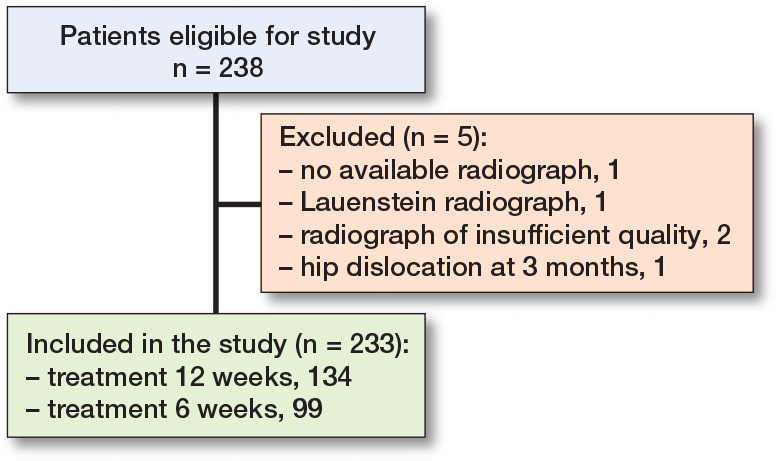
Flowchart of included patients.

The treatment length was 6 weeks for 99 children and 12 weeks for 134 children. No patients with a diagnosed syndrome were included in the study. We had to exclude 5 patients in the present study: 1 patient without available radiograph at 3 months, 1 patient with only a Lauenstein radiograph at 3 months, 2 due to radiographs of insufficient quality at 3 months, and 1 due to a hip dislocation at 3 months (Figure 2).

This leaves us with 228 patients and 304 pathologic hips, 76 bilateral and 152 unilateral, of which 102 (67%) were in the left hip (Table 1).

**Table 1:** Number of patients in each group according to treatment length, separated by hip pathology

Treatment	Patients	Healthy hips	Pathologic hips				
			Bilateral (pairs)	Left	Unilateral Right	Total	All
6 weeks	96	67	29	41	26	67	125
12 weeks	132	85	47	61	24	85	179
All	228	152	76	102	50	152	304

### Correlation between AI at 3 and 12 months of age (Table 2)

**Figure 3:**
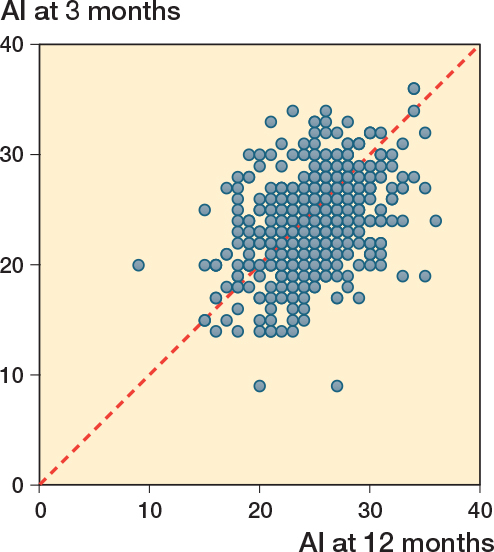
Scatterplot of all hips (456 including 152 healthy hips) shows a moderate correlation of acebatular index between 3 and 12 months: r 0.43 (CI 0.35–0.50).

The correlation between AI at 3 and 12 months of age for the whole cohort was moderate: r = 0.43 (CI 0.35–0.50), P < 0.001 and the mean difference in AI was 1.0° (CI 0.6–1.3), P < 0.001 (Figure 3).

**Table 2:** Mean difference between AI at 3 and 12 months and Pearson correlation on the difference with 95% confidence intervals

Hips/group	n	Mean difference (CI)	P value	Pearson r (CI)	P value
All	456	1.0 (0.6 to 1.3)	< 0.001	0.43 (0.35–0.50)	< 0.001
Healthy	152	0.7 (–0.1 to 1.5)	0.04	0.32 (0.17–0.46)	< 0.001
Pathologic	304	1.1 (0.6 to 1.6)	< 0.001	0.48 (0.39–0.56)	< 0.001
Treated for					
12 weeks	264	2.2 (1.7 to 2.8)	< 0.001	0.47 (0.37–0.56)	< 0.001
6 weeks	192	–0.75 (–1.4 to –0.12)	0.02	0.46 (0.34–0.56)	< 0.001

The correlation for hips treated for 12 weeks was moderate: 0.47 (CI 0.37–0.56), P < 0.001 and the mean difference was 2.2° (CI 1.7–2.8), P < 0.001.

The correlation for hips treated for 6 weeks was moderate: 0.46 (0.34–0.56), P < 0.001 and the mean difference was –0.75° (CI –1.4 to –0.12), P = 0.02.

**Figure 4:**
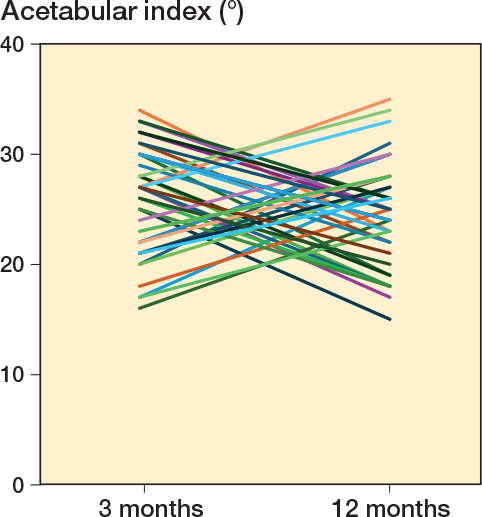
The 40 hips with largest change in acetabular index between 3 and 12 months in hips treated for 6 weeks.

Individual changes in AI differed widely, from a large increase to a large decrease (Figure 4).

### Mean AI at different time points (Table 3)

**Figure 5:**
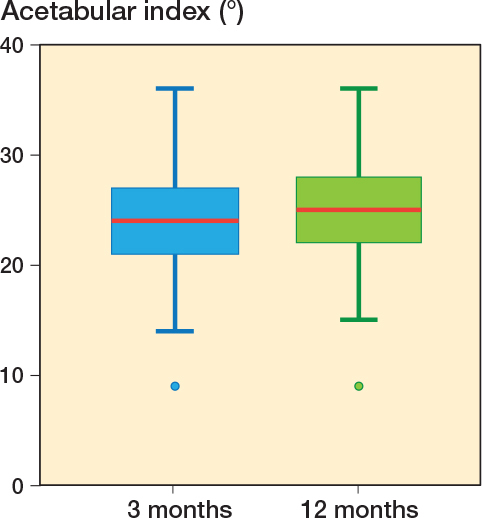
Boxplot with acetabular index at 3 and 12 months of all hips including healthy hips. Red line = mean, box = 25th–75th percentile, whisker = minimum/maximum, dots = outliers.

The mean AI for the whole cohort was 23.9° (CI 23.5–24.3) at 3 months and 24.9° (CI 24.6–25.3) at 12 months (Figure 5).

**Table 3:** Mean AI with 95% confidence interval in each group according to treatment length, separated by hip pathology, measured at 3 and 12 months

Treatment	Mean AI (CI) at 3 months			Mean AI (CI) at 12 months		
	Healthy	Pathologic	All	Healthy	Pathologic	All
6 weeks	25.3 (24.3–26.4)	25.7 (25.0–26.4)	25.5 (24.9–26.1)	24.1 (23.2–25.0)	25.2 (24.4–26.0)	24.8 (24.2–25.4)
12 weeks	22.5 (21.7–23.4)	22.9 (22.3–23.6)	22.8 (22.3–23.3)	24.7 (23.8–25.6)	25.3 (24.6–25.7)	25.0 (24.5–25.5)
Both groups	23.8 (23.1–24.4)	24.0 (23.5–24.6)	23.9 (23.5–24.3)	24.4 (23.8–25.1)	25.2 (24.7–25.6)	24.9 (24.6–25.3)

In hips treated for 6 weeks, the mean AI was 25.5° (CI 24.9–26.1) at 3 months and 24.8° (CI 24.2–25.4) at 12 months.

In hips treated for 12 weeks, the mean AI was 22.8° (CI 22.3–23.3) at 3 months and 25.0° (CI 24.5–25.5) at 12 months.

### Intra-observer analysis

A 2-way random-effects model ICC with absolute agreement, calculated on AI measured by AS on 192 hips from the 6-week group, showed good reliability with an ICC of 0.84 (CI 0.65–0.78).

## Discussion

We aimed to examine the correlation between acetabular index (AI) at 3 and 12 months. We found a moderate correlation between AI at 3 and 12 months with non-clinical differences for both 6 and 12 months of treatment.

Interestingly, 12 weeks of treatment resulted in lower AI at 3 months compared with AI at 3 months after 6 weeks of treatment. The AI at 3 months in children treated for 12 weeks was actually even lower than has been reported in the normal population at this age (AI 24–26) [4,9,12,13]. This difference had, however, disappeared at 12 months of age. Our findings indicate that there are factors other than the anatomical appearance (AI at 3 months) that determine future hip dysplasia.

Although subtle, we found an unexpected increase in AI between 3 and 12 months in the 12-week group (see Tables 2 and 3). As the 12-week cohort was larger than the 6-week cohort, the low AI at 3 months in that subgroup affects the cohort as a whole and can give a misleading interpertation that AI in general increases between 3 and 12 months. We do not think this is the case as AI decreased as expected in the 6-week group (see Table 2).

Previous studies of healthy hips have reported AI values at 3 months age of 24–26°, and 22–23° at 12 months [4,9,12,13]. Our patients’ pathologic hips with a mean AI of 25° at 12 months were more “dysplastic” compared with the general population. This was expected and is supported by previous studies [9]. However, the contralateral “healthy” hips similarly demonstrated a mean AI of 24°, which is higher than the normal population. This finding indicates that DDH is a general disease and affects stable hips as well. We did not include healthy controls in this study, and are aware that the contralateral hip cannot be seen as fully normal, both as it was contralateral to a dislocated hip and because it had been treated in the von Rosen splint during its early development.

Our findings may have implications for both treatment and follow-up of patients with DDH. Since 2021, all abduction treatment for infant DDH is reported to the Swedish Paediatric Orthopaedic Quality Register (SPOQ). In that register, type of treatment, treatment length, and AI at age 18 months is reported [14]. In addition to the compulsory pelvis radiograph at 18 months, all children with DDH at Skåne University Hospital, as well as referred cases with suspected instability, are investigated with a pelvis radiograph at age 3 months. The main purpose of that investigation is to identify the rare cases of late-detected hip dislocation. However, we also measure the AI and patients with high AI (typically > 30°) may receive additional abduction treatment and further radiographic controls before the age of 18 months. This regimen must be questioned, as our findings do not support a strong correlation between AI at 3 and 12 months of age. This may also explain why countries with universal ultrasound screening according to Graf report higher rates of late-detected DDH compared with Sweden, where emphasis is put on clinical screening of instability [15-17]. The α-angle on static ultrasound is similar to the AI on radiographs. Hence, the angle of the acetabular roof (reflected by AI and the Graf α-angle) in small children (3 months) is variable and probably not a good predictor of later hip dysplasia.

### Limitations

A major limitation is that this study lacks a control group of healthy children. It would be ethically questionable to perform pelvic radiographs in asymptomatic children. Instead, we must compare our data with previous studies of healthy hips [9,12]. As all measurements were made by the first author, we cannot rule out some degree of measurement error despite a good ICC value. However, the inter- and intra-observer reliability when analyzing AI is high [4,18,19].

### Conclusion

The correlation between the acetabular index at 3 and 12 months was moderate, with non-clinical differences for both 6 and 12 weeks of treatment. The small increase in mean AI was most likely explained by a low AI at 3 months after 12 weeks of treatment.

*In perspective*, given the only moderate correlation between AI at 3 and 12 months, we believe that AI measurements at 3 months are not useful to predict later acetabular dysplasia. We propose that the radiographic control at 3 months remains to catch treatment failures such as hip dislocations, but not to guide additional treatment based on AI at that time point. If a static ultrasound is the preferred method of detecting a dysplastic hip, the same conclusion may also be valid for the Graf α-angle.

## Notes

Adam Sand performed radiographic measurements and wrote the first manuscript draft. All authors contributed to study design, analysis, interpretation of data, and critical review of the manuscript.

The authors would like to thank Aleksandra Turkiewicz for statistical support.

Handling co-editors: Ilkka Helenius and Robin Christensen

*Acta* thanks Nanni Allington, Peter John Cundy and Martin Gottliebsen for help with peer review of this manuscript.
